# Clinical utility of a serum biomarker panel in distinguishing prostate cancer from benign prostate hyperplasia

**DOI:** 10.1038/s41598-021-94438-4

**Published:** 2021-07-23

**Authors:** Michael A. Kiebish, Poornima Tekumalla, Shobha Ravipaty, Albert Dobi, Shiv Srivastava, Wenfang Wu, Saurabh Patil, Tracey Friss, Allison Klotz, Alagarsamy Srinivasan, Jennifer Cullen, Inger L. Rosner, Amina Ali, Sandra Laszlo, Michele Petrovic, Neil Fleshner, Jeonifer Garren, Greg Miller, Nischal Mahaveer Chand, Leonardo O. Rodrigues, Elder Granger, Mark D. Kellogg, Shen Luan, Eleftherios Diamandis, Viatcheslav R. Akmaev, Rangaprasad Sarangarajan, Chas Bountra, Stephen J. Freedland, David G. McLeod, Niven R. Narain

**Affiliations:** 1 BERG, 500 Old Connecticut Path, Building B, Framingham, MA 01701 USA; 2 Center for Prostate Disease Research, John P. Murtha Cancer Center Research Program, Department of Surgery, Uniformed Services University of the Health Sciences and the Walter Reed National Military Medical Center, Bethesda, MD 20817 USA; 3 Henry M. Jackson Foundation for the Advancement of Military Medicine, Bethesda, MD 20817 USA; 4 University Health Network, Toronto, ON M5G 2C4 Canada; 5 Department of Laboratory Medicine and Pathology, Harvard Medical School, Boston Children’s Hospital, Boston, MA 02115 USA; 6 Mount Sinai Hospital, Toronto, ON M5T 3L9 Canada; 7 Department of Clinical Medicine, University of Oxford, Oxford, OX3 7LF UK; 8 Center for Integrated Research in Cancer and Lifestyle, Cedar-Sinai, Los Angeles, CA 90048 USA; 9 Durham VA Medical Center, Durham, NC 27705 USA; 10 Present Address: Department of Biochemistry and Molecular and Cell Biology, Georgetown University School of Medicine, Washington, DC 20057 USA; 11 Present Address: Nano Bio Diagnostics LLC, West Chester, PA 19382 USA; 12 Present Address: Case Comprehensive Cancer Center, Cleveland, OH 44106 USA; 13 Present Address: Inova Health System, Fairfax, VA 22031 USA

**Keywords:** Biomarkers, Oncology, Urology

## Abstract

Prostate-specific antigen (PSA) screening for prostate cancer (PCa) is limited by the lack of specificity but is further complicated in the benign prostatic hyperplasia (BPH) population which also exhibit elevated PSA, representing a clear unmet need to distinguish BPH from PCa. Herein, we evaluated the utility of FLNA IP-MRM, age, and prostate volume to stratify men with BPH from those with PCa. Diagnostic performance of the biomarker panel was better than PSA alone in discriminating patients with negative biopsy from those with PCa, as well as those who have had multiple prior biopsies (AUC 0.75 and 0.87 compared to AUC of PSA alone 0.55 and 0.57 for patients who have had single compared to multiple negative biopsies, respectively). Of interest, in patients with PCa, the panel demonstrated improved performance than PSA alone in those with Gleason scores of 5–7 (AUC 0.76 vs. 0.56) and Gleason scores of 8–10 (AUC 0.74 vs. 0.47). With Gleason scores (8–10), the negative predictive value of the panel is 0.97, indicating potential to limit false negatives in aggressive cancers. Together, these data demonstrate the ability of the biomarker panel to perform better than PSA alone in men with BPH, thus preventing unnecessary biopsies.

## Introduction

Prostate cancer, owing to both its incidence and associated mortality, is an important public health problem. For this reason, as well as several others, screening for prostate cancer is both desirable and feasible. In spite of many issues, widespread adoption of prostate cancer screening using prostate specific antigen (PSA) testing has resulted in approximately 40% decreases in prostate cancer mortality from an epidemiologic perspective^1^, with approximately 45–70% of the decline attributable to PSA-based prostate cancer screening^2,3^. Unfortunately, currently adopted screening approaches utilizing PSA testing, may not be suitable^4^, as a result of significant harm due to medical evaluation including biopsy, over-diagnosis and overtreatment^5^. Elevated PSA lacks both sensitivity and specificity for the diagnosis of clinically significant prostate cancer: while a significant proportion of patients with an elevated PSA, regardless of definition, will not have prostate cancer, up to 25% of men aged 50 to 70 years old with a normal PSA would be expected to have high grade prostate cancer^6^.

The lack of specificity of prostate cancer screening using PSA testing is driven in large part by the co-incidence of benign prostate hyperplasia (BPH), a condition which can also result in increased PSA levels, in this patient population^7^. Consequently, there is a critical unmet need for improvement in distinguishing patients with BPH from patients with PCa. Several new screening tests have been developed including measuring PSA derivatives^8,9^, magnetic resonance imaging (MRI) to detect abnormal prostates for further testing^10^, ELISA-based screens^11^, and multi-analyte tests such as the STHLM3, which measures a combination of plasma protein markers, genetic polymorphisms, clinical variables, and PSA levels^12^. Additionally, there are several commercially available tests including the K4Score test, which has been tested in Europe and the U.S., and can discriminate between high-risk and low-risk disease^13^ as well as the prostate health index (PHI) test that can also discriminate between high-risk and low-risk cancers, but may not be able to accurately predict disease severity when challenged with intermediate PSA values^14–16^. However, none of them have been extensively evaluated in men with BPH.

In the current study, we assessed the combinatorial utility of filamin-A (FLNA), age, and prostate volume, in predicting PCa risk in a cohort of men enriched with BPH. FLNA has been shown to be influenced by androgens impacting cell migration, FLNA cleavage, and intracellular signaling. Thus, FLNA demonstrates mechanistic biological insight into its potential pathophysiological use as a marker in serum^17,18^. Combined with a demographic risk factor (age) and clinical factor such as prostate volume, this integrated assessment was evaluated in 300 men with clinically diagnosed BPH and confirmed negative biopsy for prostate cancer [either single or multiple biopsies (2 to 4)] compared to 477 men with biopsy confirmed PCa. The clinical design was structured to evaluate men with PSA between 4 and 10 ng/mL, with a negative digital rectal exam (DRE) to demonstrate the utility of the marker panel stratifying men with BPH (that had undergone one or multiple biopsies) from men with confirmed negative biopsy compared to those with confirmed PCa.

## Materials and methods

### Clinical population

**Figure 1:**
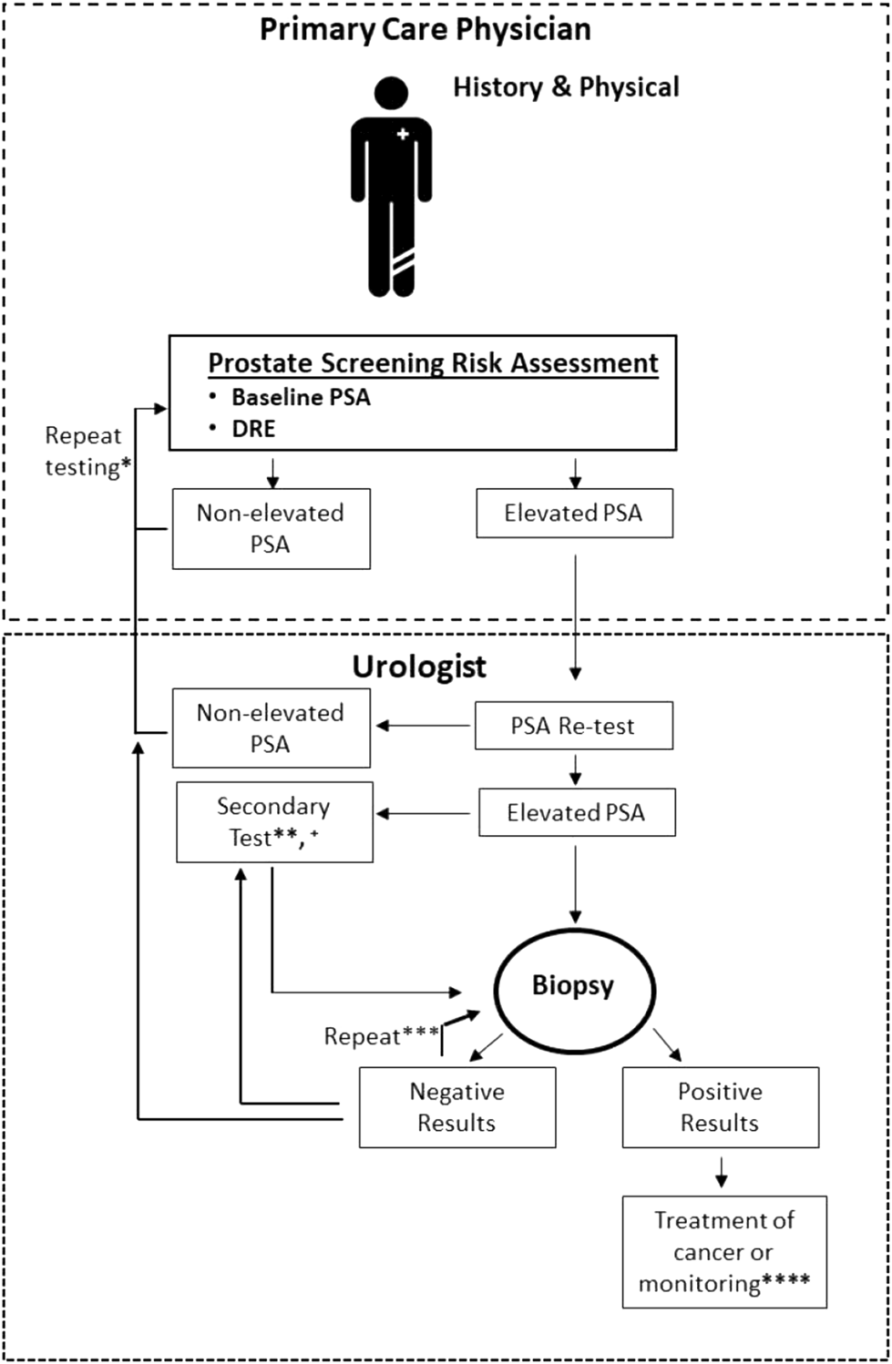
Current diagnostic paradigm for prostate cancer diagnosis and biopsy.

Serum samples were obtained prior to biopsy from retrospective (Durham Veteran Affairs Medical Center, CPDR/Walter Reed, University of Toronto/UHN) biobanks as well as a prospective trial (Cleveland Clinic) under IRB approval. Ethnic diversity of cohorts as well as their demographic features are reported in Table 1. For retrospective samples, selection criteria were patients selected based on a negative DRE, AUA symptom scores between 8 and 21 (where info was available), and PSA between 4 and 10 ng/mL. PSA measurements across sites were performed on the Cobas E602 platform (Roche Diagnostics) using the Elecsys Total PSA (Detection limits LoB 0.006 ng/mL, LoD 0.010 ng/mL, LoQ 0.014 ng/mL and measuring range 0.006–100 ng/mL) as well as the Hybritech platform for total PSA (measuring range 0.1–150.0 ng/mL). Prostate volume was measured using transrectal ultrasonography (TRUS). For negative cases, patients were classified for BPH or lower urinary tract symptoms (LUTS) based on symptom score, pathology, and prostate volume. For CPDR/Walter Reed retrospective cohort, confirmed BPH patients were longitudinally followed and results of serial negative biopsies (2–4 biopsies—in 95 of the patients) were assessed in patients with serum collected prior to the first biopsy. The present study was designed to evaluate several clinical endpoints demonstrating its clinical utility in men with BPH or PCA (Fig. 1), which could influence its utility in a clinical setting (Fig. 1).

**Table 1:** Patient demographics of 777 patients evaluated.

	BPH/LUTS			PCa		
	Mean	±	SD	Mean	±	SD
Age	64.6	±	8.5	61.9	±	8
PSA	6.7	±	4.7	6.5	±	5.9
Prostate volume	61.4	±	27.3	42.3	±	26.4
**Gleason**						
5–7					404	
8–10					39	

	n	% Total		n	% Total	
**Race**						
Caucasian-American	191	64%		281	59%	
African-American	48	16%		139	29%	
Other	61	20%		57	12%	
**Clinical site**						
CPDR/Walter Reed	119	40%		239	50%	
Veteran Affairs	58	19%		34	7%	
UHN	118	39%		78	16%	
CCF	5	2%		126	26%	
Total	300			477		

Age (years), PSA levels (ng/mL), and prostate volume (mL) (Mean ± standard deviation, SD). Distribution of patients by race and sampling distribution of patients from various clinical sites.

### Human rights

All procedures performed in studies involving human participants were in accordance with the ethical standards of the institutional and/or national research committee and with the 1964 Helsinki declaration, and its later amendments or comparable ethical standards. All studies had an approved IRB from Cleveland Clinic, Veteran Affairs Durham, University Hospital Network (UHN), and Uniformed Services University/Walter Reed National Medical Center for sample collection and usage. Informed consent was obtained from all participants.

### Quantitation of FLNA peptides by immunoprecipitation and LC–MS/MS (MRM) analysis

#### Antibody immobilization

Three mouse monoclonal antibodies, Anti-FLNA 2C12, Anti-FLNA 3F4, and Anti-FLNA 6E3 were immobilized onto an agarose support using the Thermo Fisher Scientific Pierce Direct IP Kit (Thermo Fisher Scientific) according to the manufacturer’s protocol, with a few modifications as previously described in Ravipaty et al. 2017^19^. 200 µg of each of the three antibodies, were coupled individually to 200 µL of AminoLink Plus coupling resin and stored at 4 °C until needed.

#### Immunoprecipitation and preparation of calibration standards

Immunoprecipitation was performed using the Pierce Direct IP Kit (Thermo Fisher Scientific) according to the manufacturer’s protocol with few modifications. Immunoprecipitation tubes were prepared by aliquoting 5 µL of each of the three antibody-coupled resins into the IP tube (Pierce Direct IP Kit, Thermo Fisher Scientific). The resin was washed twice with 200 µL of IP lysis/wash buffer. 100 µL of human serum sample or 100 µL of water (surrogate matrix) was added to each IP tube along with 500 µL of prepared lysis buffer solution (IP lysis/wash buffer with 1.2X Halt protease cocktail inhibitor; Thermo Fisher Scientific) and 0.5 M EDTA, then incubated overnight at 4 °C with end-over-end mixing. The resin was washed five times with 200 µL of IP lysis/wash buffer and once with 100 µL of 1X conditioning buffer. The captured proteins were eluted with 50 µL of elution buffer with an incubation time of 15 min, and then neutralized with 5 µL of 1 M Tris HCl, pH 9.0 (Teknova, Hollister, CA). The IP eluates from the surrogate matrix were used to prepare P2 (AGVAPLQV) peptide calibration curves by spiking with a P2 synthetic peptide (Genscript, Piscataway, NJ) stock solution (0.2/0.36 µg/mL) followed by serial dilution. P2 calibration standards ranged from 125 to 2000 pg/mL. All samples were then subjected to trypsin digestion as described below and as previously described in Ravipaty et al. 2017^19^.

#### Digestion of IP-extracted samples using trypsin

Trypsin digestion was performed using the Flash Digest Kit (Perfinity Biosciences, West Lafayette, IN) following the manufacturer’s protocol with few modifications. Flash digest tubes were equilibrated to room temperature, and then centrifuged for 1 min at 1500 × *g* and 5 °C. 50 µL of each sample, 25 µL of digestion buffer (Perfinity Biosciences), and 5 µL of working internal standard (Thermo Fisher Scientific) solution (P2/P4 10/30 ng/mL) were added to the Flash digest tubes. After vortexing, samples were digested at 70 °C for 20 min in the Eppendorf, Thermo Mixer C (Eppendorf). The Flash digest tubes were then centrifuged for 5 min at 1500 × *g* and 5 °C. A 60 µL aliquot of the supernatant was transferred to an LC–MS vial.

#### LC–MS/MS (MRM) analysis

MRM analyses were performed on a 6500 QTRAP mass spectrometer (SCIEX) equipped with an electrospray source, a 1290 Infinity UPLC system (Agilent Technologies, Santa Clara, CA), and a XBridge Peptide BEH300 C18 (3.5 μm, 2.1 mm × 150 mm) column (Waters, Milford, MA). Liquid chromatography was carried out at a flow rate of 400 µL/min, and the sample injection volume was 30 µL. The column was maintained at a temperature of 60 °C. Mobile phase A consisted of 0.1% formic acid (Sigma Aldrich) in water (Thermo Fisher Scientific), and mobile phase B consisted of 0.1% formic acid in acetonitrile (Thermo Fisher Scientific). The gradient with respect to %B was as follows: 0–1.5 min, 5%; 1.5–2 min, 5–15%; 2–5 min, 15%; 5–7.1 min, 15–20%; 7.1–8.1 min, 20–80%; 8.1–9.0 min, 80%; and 9.0–9.1 min, 80–5%. 9.1–16 min, 5%. The instrument parameters for 6500 QTRAP mass spectrometer were as follows: Ion spray voltage of 5500 V, curtain gas of 20 psi, collision gas set to “medium”, interface heater temperature of 400 °C, nebulizer gas (GS1) of 80 psi and ion source gas (GS2) of 80 psi, and unit resolution for both Q1 and Q3 quadrupoles as previously described in Ravipaty et al. 2017^19^.

### IPMRM data analysis and quantitation

Data analysis was performed using the Analyst® software (version 1.6.2, SCIEX Framingham, MA) and peak integrations were reviewed manually. The calibration curve for FLNA P2 peptide was constructed by plotting the peak area ratios (analyte/internal standard) versus concentration of the standard with 1/ × 2 linear least square regression. The regression equations from P2 calibration standards were used to back-calculate the measured P2 concentrations for each QC and unknown sample.

### Statistical analysis

Logistic Regression models were built and compared for their ability to classify patients with PCa with Gleason score (≤ 7), Gleason score (≥ 8), and absence of cancer on biopsy. Area under the curves for comparisons were determined by the sensitivity and specificity of panel predictability. The resulting PCa panel predictive algorithms were based on the regression models and probability threshold values selected to achieve a certain level of test sensitivity or specificity. All analyses were performed in R 3.2.2 with a significance level of 0.05, unless otherwise stated.

## Results

**Figure 2:**
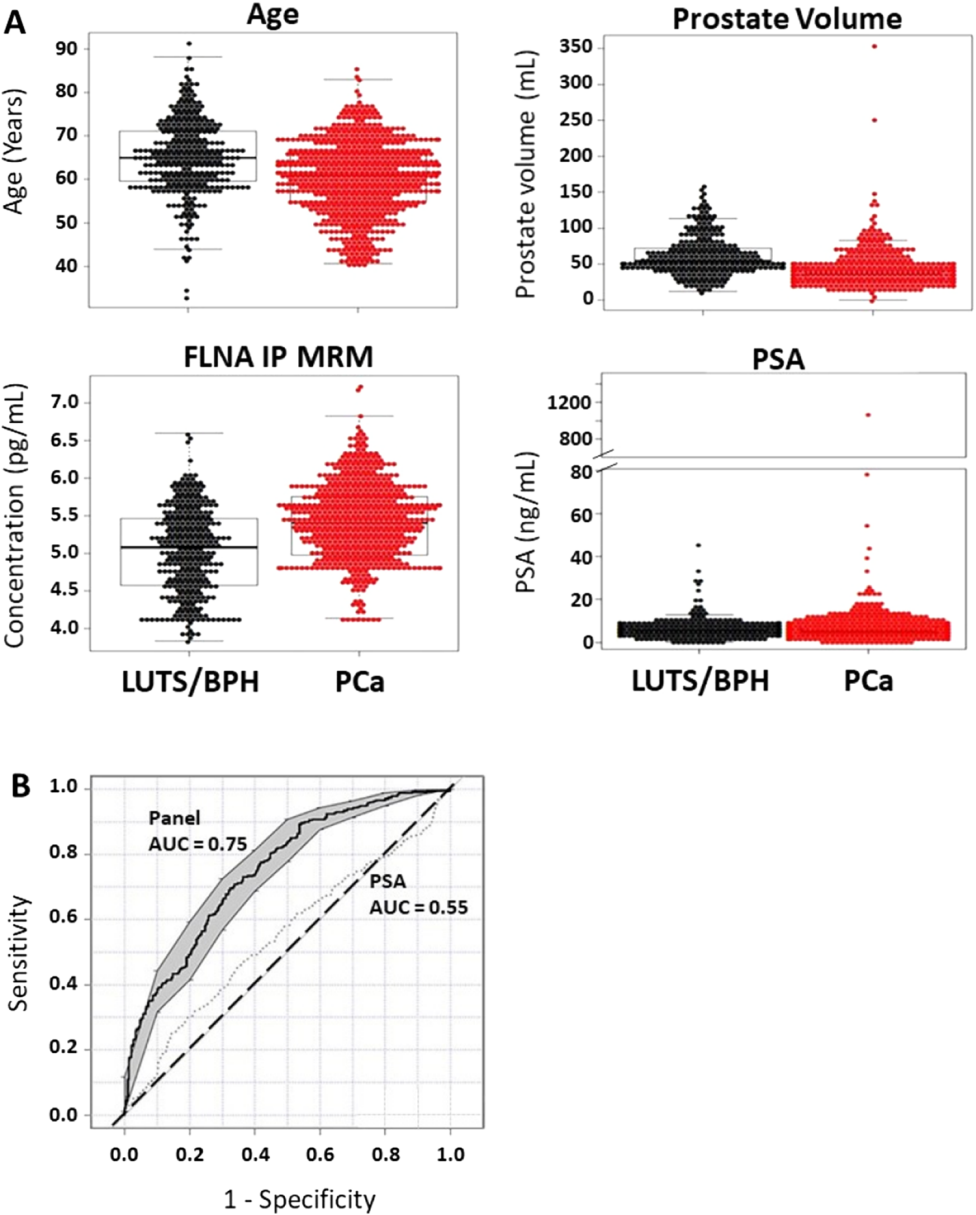
Prostate Cancer Biomarker Panel Performs Better Than PSA Alone in Differentiating Patients with Lower Urinary Tract Symptoms (LUTS) or Benign Prostate Hyperplasia (BPH) from Prostate Cancer (PCa). (**A**) Beeswarm and boxplot graphs showing median, interquartile range and distribution of age (years), prostate volume (mL), serum FLNA concentrations (pg/mL), and serum PSA concentrations (ng/mL) from patients with LUTS/BPH compared to those with PCa. Data represents n = 300 LUTS/BPH and n = 477 PCa. (**B**) Receiver operator characteristics (ROC) curve for prostate cancer biomarker panel demonstrates better performance in differentiating patients with LUTS/BPH from PCa compared to PSA alone. Area under the curve (AUC) for the panel is 0.75 versus PSA alone 0.55. Shaded grey regions indicate standard error.

Regression modeling analysis was utilized to identify the optimal set of predictive factors for identification of men with BPH compared to those with confirmed PCa. The combination of the factors age, prostate volume, and FLNA was found to have better predicative performance than PSA alone in discriminating LUTS/BPH from PCa (AUC 0.75 vs. 0.55; Fig. 2, Table 2**)**. This resulted in a cutoff for the model at 0.498 and yielded a specificity of 0.45 with positive and negative predictive values of 0.72 and 0.74, respectively (Table 2). The diagnostic odds ratio for the biomarker panel in predicting PCa was 7.4 (95% CI 4.9–11). Moreover, the performance of the biomarker panel indicated that in the current study cohort (n = 777 patients), 130 (43%) patients without PCa would not be recommended for biopsy, reducing the number of unnecessary biopsies compared to all 300 patients with PSA alone who would have been recommended for a biopsy. Additionally, we performed a comparison with PSA and clinical variables using decision curve and statistic analysis, which demonstrated improved statistical performance using FLNA, prostate volume and age compared to PSA, prostate volume and age (Supplemental Fig. 1A–E). FLNA, prostate volume and age assessment was also performed across sites, demonstrating statistical significance (Supplemental Fig. 1F). In comparison, utility of PSA alone across the 4 different sites did not demonstrate diagnostic utility or statistical significance (Supplemental Fig. 1G).

**Table 2:** Diagnostic performance in BPH/LUTS patients undergoing a biopsy or multiple biopsies.

Model	AUC	Sensitivity	Specificity	PPV	NPV	OR	(CI)
Biopsy	0.75	0.9	0.45	0.72	0.74	7.4	(4.9,11)*
Multiple biopsy	0.87	0.9	0.67	0.93	0.58	18.7	(10.8,33)*

Diagnostic performance (AUC), sensitivity, specificity, positive predictive value (PPV) and negative predictive value (NPV), odds ratio (OR), and 95% confidence interval (CI) of the OR for each model. *p < 0.05 in discrimination accuracy.

**Figure 3:**
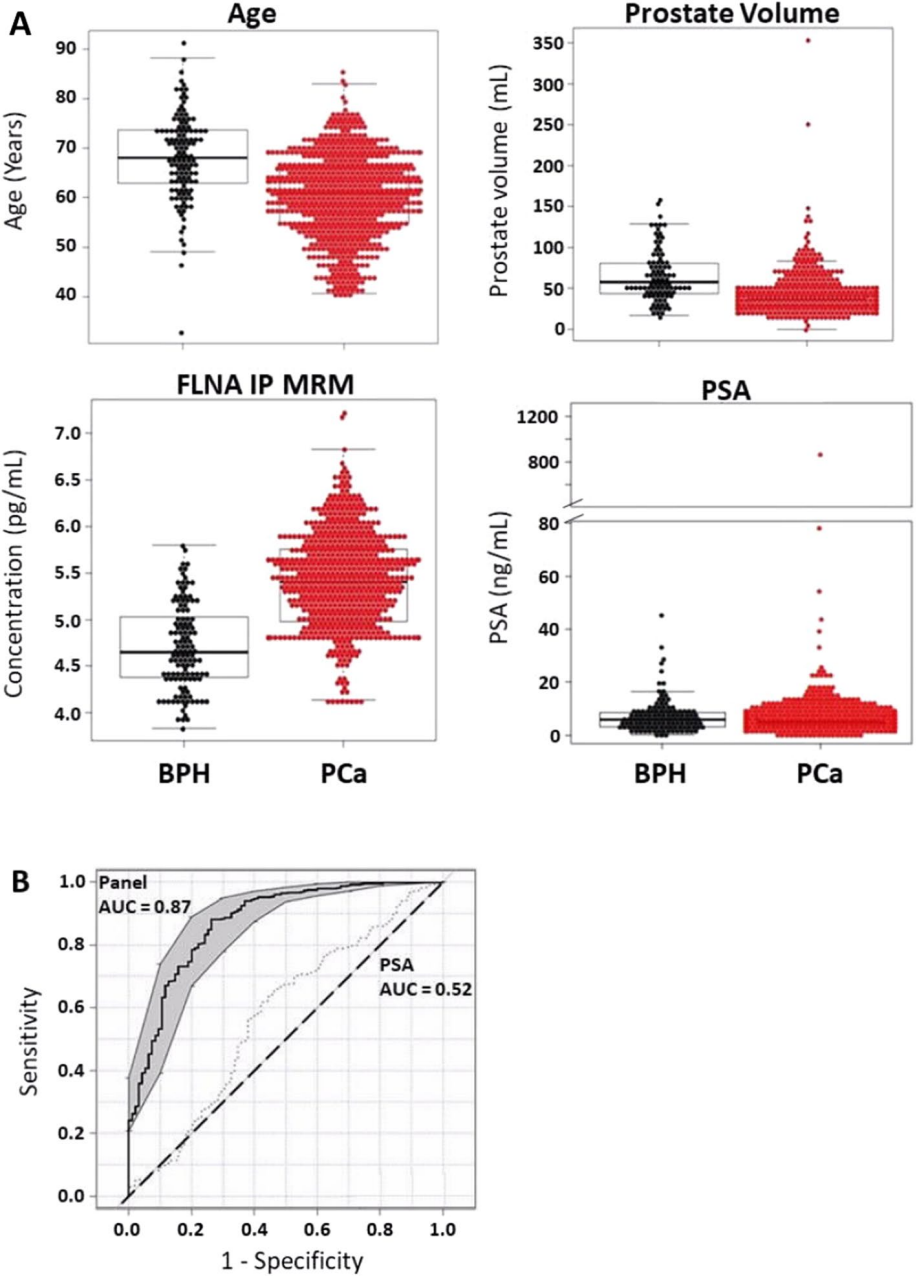
Prostate Cancer Biomarker Panel Differentiates Lower Urinary Tract Symptoms/Benign Prostate Hyperplasia (LUTS/BPH) from Prostate Cancer (PCa) in Patients who have had multiple biopsies. (**A**) Beeswarm and boxplot graphs showing median, interquartile range and distribution of age (years), prostate volume (mL), serum FLNA concentrations (pg/mL), and serum PSA concentrations (ng/mL) from patients who have had multiple biopsies. Data represents n = 94 LUTS/BPH and n = 477 PCa. (**B**) Receiver operator characteristics (ROC) curve for prostate cancer biomarker panel demonstrates better performance in differentiating patients with LUTS/BPH from PCa compared to PSA alone. Area under the curve (AUC) for the panel is 0.87 versus PSA alone 0.57. Shaded grey regions indicate standard error.

Next, we evaluated the panel in 95 men with LUTS/BPH that had undergone multiple biopsies evaluating their first serum sample prior to the first biopsy to determine the performance of the panel preventing unnecessary biopsies. For each of the factors in the biomarker panel, differences were observed between patients with LUTS/BPH and PCa in patients who have had more than one biopsy (Fig. 3A). However, when the factors are combined (i.e. age, prostate volume, and FLNA levels) this yielded a diagnostic performance that is improved over that of PSA alone in discriminating patients with LUTS/BPH from PCa in patients who have had multiple biopsies (AUC 0.87 vs. 0.52; Fig. 3B; Table 2). The diagnostic OR of the biomarker panel in patients who had multiple biopsies was 18.7 (95% CI 10.8–33, Table 2). Thus, compared to PSA alone, the biomarker panel would reduce the number of biopsy recommendations by 67% in patients without PCa. These findings suggest that the use of the biomarker panel prior to biopsy improves the selection of men for biopsy, in addition to reducing the need for biopsy and unnecessary harm from intervention in patients with LUTS/BPH. Decision curve and statistical assessment was performed for FLNA, prostate volume, and age compared to PSA, prostate volume, and age. FLNA plus clinical variables demonstrated a significant improvement in AUC (0.87 vs. 0.75), PPV (0.93 vs. 0.88), NPV (0.58 vs. 0.43), OR (18.7 vs. 5.56) and *p*-value (4.2 E−31 vs. 2.0 E−10) compared to PSA, prostate volume, and age, which provides additional evidence for the utility of the FLNA panel in preventing multiple unnecessary biopsies (Supplemental Fig. 2A–E).

**Figure 4:**
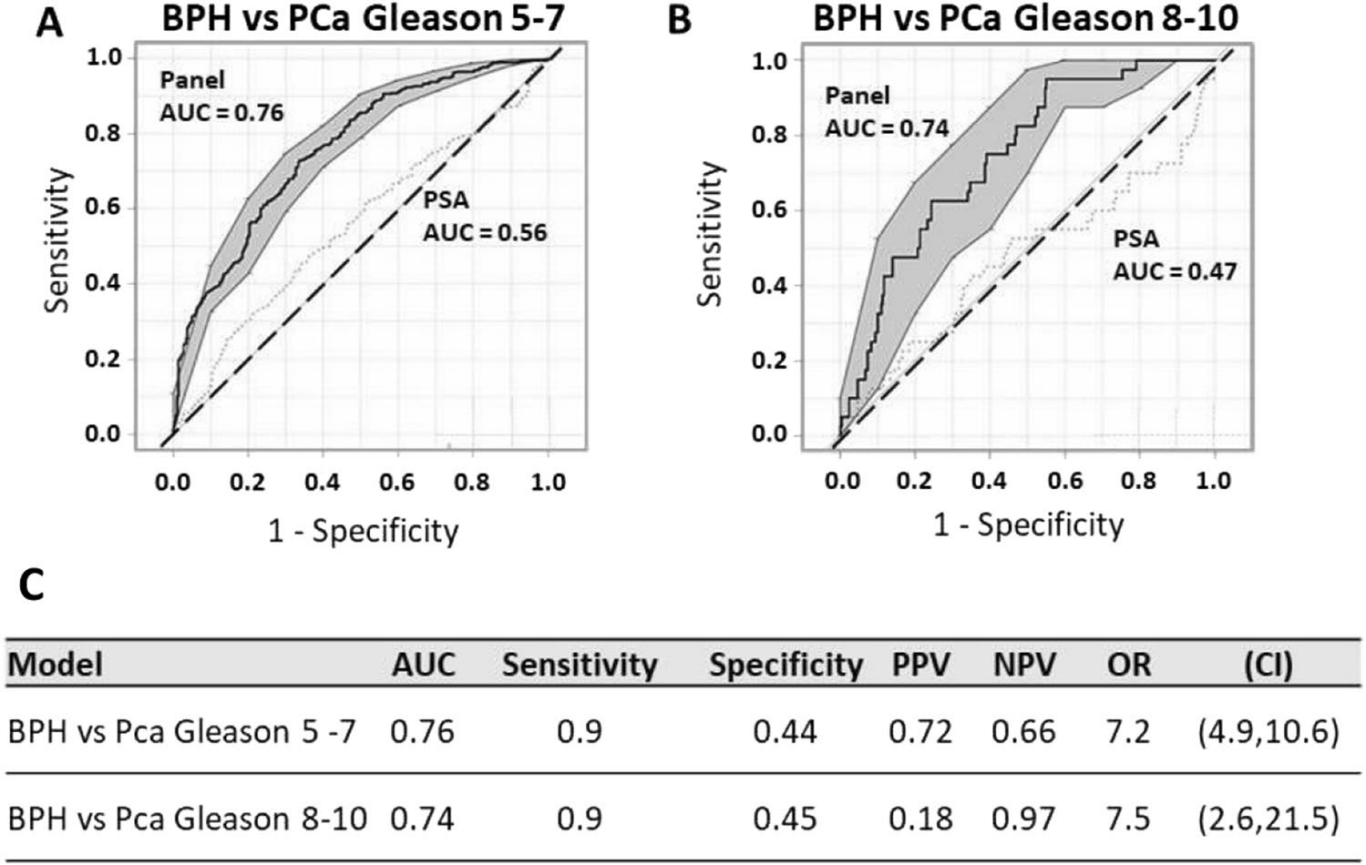
Prostate Cancer Biomarker Panel Differentiates Lower Urinary Tract Symptoms/Benign Prostate Hyperplasia (LUTS/BPH) from Prostate Cancer (PCa) in Patients with Intermediate and High Gleason Score. (**A**) Receiver operator characteristics (ROC) curve for prostate cancer biomarker panel demonstrates better performance in differentiating patients with LUTS/BPH from PCa compared to PSA alone in patients with an intermediate Gleason score (5–7). Area under the curve (AUC) for the panel is 0.76 versus PSA alone 0.56. Shaded grey regions indicate standard error. (**B**) Receiver operator characteristics (ROC) curve for prostate cancer biomarker panel demonstrates better performance in differentiating patients with LUTS/BPH from PCa compared to PSA alone in patients with a high Gleason score (8–10). Area under the curve (AUC) for the panel is 0.74 versus PSA alone 0.47. Shaded grey regions indicate standard error. (**C**) Diagnostic performance (AUC), sensitivity, specificity, positive predictive value (PPV), negative predictive value (NPV), odds ratio (OR), and 95% confidence interval (CI) of the OR for each model.

We next assessed the biomarker panel’s ability to discriminate LUTS/BPH from PCa in patients with a Gleason score (5–7), and those with Gleason score (≥ 8). Results indicate a diagnostic performance that is improved over that of PSA alone in discriminating patients with LUTS/BPH from PCa in patients with Gleason score (5–7; AUC 0.76 vs. 0.56; Fig. 4A, C). The diagnostic Odds Ratio (OR) of the biomarker panel in patients who had biopsies is 7.2 (95% CI 4.9–10.6, Fig. 4C). Furthermore, the biomarker panel’s performance in discriminating LUTS/BPH from patients with more aggressive PCa, Gleason (≥ 8), was also determined to be significantly improved compared to PSA alone (AUC 0.74 vs. 0.47; Fig. 4B). Moreover, diagnostic sensitivity set at 0.9 yields a specificity of 0.45, and positive and negative predictive values of 0.18 and 0.97, respectively (Fig. 4C). The diagnostic OR of the biomarker panel is 7.5 (95% CI 2.6, 21.5) in discriminating patients with LUTS/BPH from those with Gleason (≥ 8) PCa (Fig. 4C). Decision curve and statistical analysis was further performed comparing FLNA versus PSA combined with the clinical variables. FLNA demonstrated improved statistical significance for Gleason (≥ 8) compared to PSA both combined with clinical variables (Supplemental Fig. 3A–E).

## Discussion

In the current study, we evaluated the clinical utility of a multiple variable prostate cancer biomarker panel test on the analysis of 777 patients assessed the combinatorial power of filamin-A (FLNA), age, and prostate volume in predicting separation of BPH versus PCa diagnoses compared to PSA. This was assessed in men undergoing a single biopsy or subsequent multiple biopsies in patients with BPH. From a health economic perspective, contributing factors related to having multiple biopsies pose a risk to the patient in the form of increased risk for infections, and the risk of impotence, among others. Patients with elevated PSA levels are often referred for a DRE and a prostate biopsy^5,20^. However, elevated PSA leads to approximately 60% of patients undergoing a negative biopsy. Men with BPH/LUTS represent a large portion of negative biopsies. Of these patients, a third will experience moderate or major side effects of a biopsy including infection, rectal bleeding, hematuria, hematospermia, lower-urinary tract symptoms, and erectile dysfunction. A small number of patients will also require hospitalization^5,21^ and rarely death can occur from sepsis.

Moreover, there is an increased and sustained cost to the patient and the healthcare system from the continued use of the PSA tests and prostate biopsies due to overdiagnosis. Medicare spent $450 million annually (2006–2009) on PSA screening and subsequent diagnostic procedures. Additionally, the cost of screening men over 75 years of age, the population least likely to benefit from the PSA test, was $145 million annually during this time period, representing a third of total Medicare spending on prostate cancer screening^22^. Current efforts focusing on the development of non-invasive biomarkers to distinguish between PCa and BPH, aggressive and indolent forms of the disease, aim to reduce the number of biopsies performed. From a physician’s perspective, it would stand to reason that a key focus on the more aggressive cancers would mitigate mortality rates, and engage more clinical vigilance on metastatic potential, which is a salient point of utility for this multiple variable test. As such, our study is a real-world analysis of a multi-modal panel versus PSA alone. One limitation of the present study is the analysis of total PSA compared to the measurement of free PSA, which has been recognized to have greater performance in PCa diagnosis. Future studies using prospectively collected patients will be evaluated to further validate the various multimodal nomograms available for these clinical comparisons.

In summary, we have demonstrated that the combination of FLNA, age, and prostate volume variables identified broad clinical utility in the potential prevention of multiple unnecessary biopsies, as well as avoidance of missing more aggressive PCa. There is a clear unmet need to avoid unnecessary biopsies in BPH/LUTS patients since PSA does not provide efficient diagnostic guidance for this patient population. In parallel, development of a test which provides guidance on ensuring that aggressive cancers are not missed and that multiple biopsies could have been avoided in BPH/LUTS patients is imperative, which is supported by the data presented.

## Supplementary Information

Supplementary Figures.
