# Supplementary material for: Clinical utility of a serum biomarker panel in distinguishing prostate cancer from benign prostate hyperplasia
Source: Sci Rep. 2021 Jul 23;11:15052. doi: 10.1038/s41598-021-94438-4 (PMC8302659; doi:10.1038/s41598-021-94438-4)
Supplement: Supplementary file 1 — Supplementary Figures. [file 41598_2021_94438_MOESM1_ESM.docx]

**Supplemental Figures 1**

**Clinical Utility of a Serum Biomarker Panel in Distinguishing Prostate Cancer from Benign Prostate Hyperplasia**

Michael A. Kiebish^1^, Poornima Tekumalla^1^, Shobha Ravipaty^1^, Albert Dobi^2,3^, Shiv Srivastava^2,4^, Wenfang Wu^1^, Saurabh Patel^1^, Tracey Friss^1^, Allison Klotz^1^, Alagarsamy Srinivasan^2,3,5^, Jennifer Cullen^2,3,6^, Inger L. Rosner^2,7^, Amina Ali^2,3^, Sandra Laszlo^8^, Michele Petrovic^8^, Neil Fleshner^8^, Jeonifer Garren^1^, Greg Miller^1^, Nischal Mahaveer Chand^1^, Leonardo Rodrigues^1^, Elder Granger^1^, Mark D. Kellogg^1,9^, Shen Luan^1^, Eleftherios Diamandis^10^, Viatcheslav R. Akmaev^1^, Rangaprasad Sarangarajan^1^, Chas Bountra^11^, Stephen Freedland^12^, David G. McLeod^2,14^, Niven R. Narain^1^

^1^BERG, Framingham, MA 01701, USA

^2^ Center for Prostate Disease Research, John P. Murtha Cancer Center Research Program, Department of Surgery, Uniformed Services University of the Health Sciences and the Walter Reed National Military Medical Center, Bethesda, MD 20817, USA

^3^ Henry M. Jackson Foundation for the Advancement of Military Medicine Bethesda, Maryland, 20817 USA

^4^ Current: Department of Biochemistry and Molecular & Cell Biology, Georgetown University School of Medicine, Washington DC, 20057, USA

^5^ Current: Nano Bio Diagnostics LLC, West Chester, PA 19382, USA

^6^ Current: Case Comprehensive Cancer Center, Cleveland, OH 44106, USA

^7^ Current: Inova Health System, Fairfax, VA 22031, USA

^8^University Health Network, Toronto, ON M5G 2C4, Canada

^9^Department of Laboratory Medicine and Pathology, Harvard Medical School, Boston Children’s Hospital, Boston, MA, 02115, USA

^10^Mount Sinai Hospital, Toronto, ON, Canada M5T 3L9, Canada

^11^Department of Clinical Medicine, University of Oxford, Oxford OX3 7LF, UK

^12^Center for Integrated Research in Cancer and Lifestyle, Cedar-Sinai, Los Angeles, CA 90048, USA

^13^Durham VA Medical Center, Durham, NC 27705, USA

^14^The co-author has passed away in December 2020

**Supplemental Figure 1**


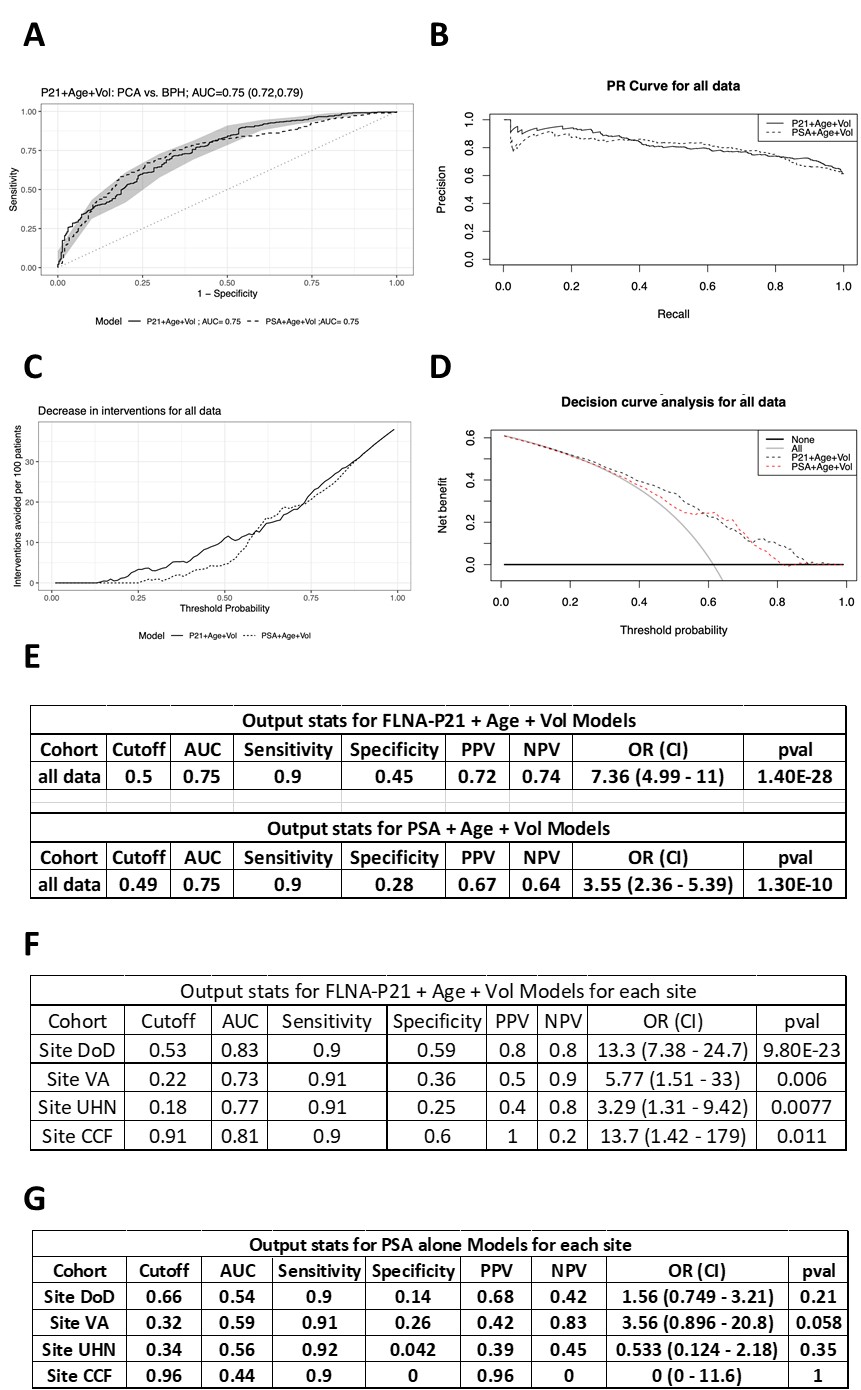


**Supplemental Figure 1:** FLNA Prostate Cancer Biomarker Panel Performs Better Than PSA combined with prostate volume and age.in Differentiating Patients with Lower Urinary Tract Symptoms (LUTS) or Benign Prostate Hyperplasia (BPH) from Prostate Cancer (PCa). **(A)** Combined ROC curve assessment. **(B)** Precision analysis, **(C)** Intervention analysis, and **(D)** decision curve analysis **(E)** statistical assessment comparing FLNA, prostate volume, and age with PSA, prostate volume, and age. **(F)** Comparison of FLNA, prostate volume and age panel across sites. **(G)** Comparison of PSA alone across sites.

**Supplemental Figure 2**

**
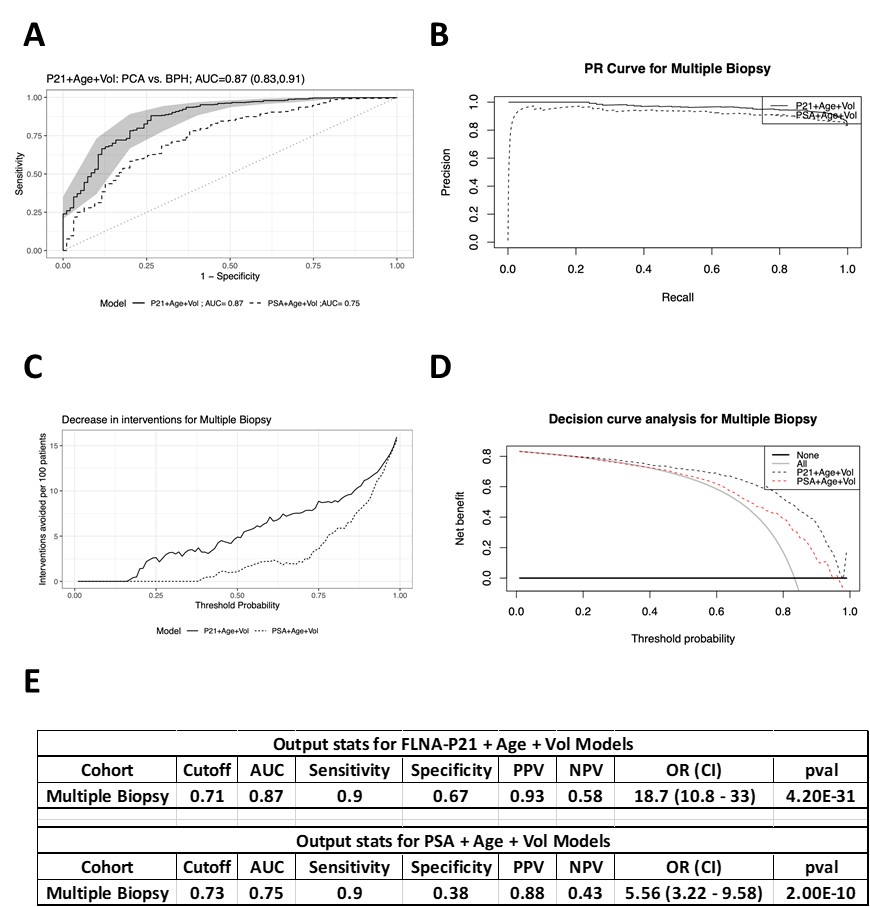
**

**Supplemental Figure 2:** FLNA Prostate Cancer Biomarker Panel Performs Better Than PSA combined with prostate volume and age.in preventing men with BPH/LUTS from having multiple biopsies. **(A)** Combined ROC curve assessment. **(B)** Precision analysis, **(C)** Intervention analysis, and **(D)** decision curve analysis **(E)** statistical assessment comparing FLNA, prostate volume, and age with PSA, prostate volume and age.

**Supplemental Figure 3**


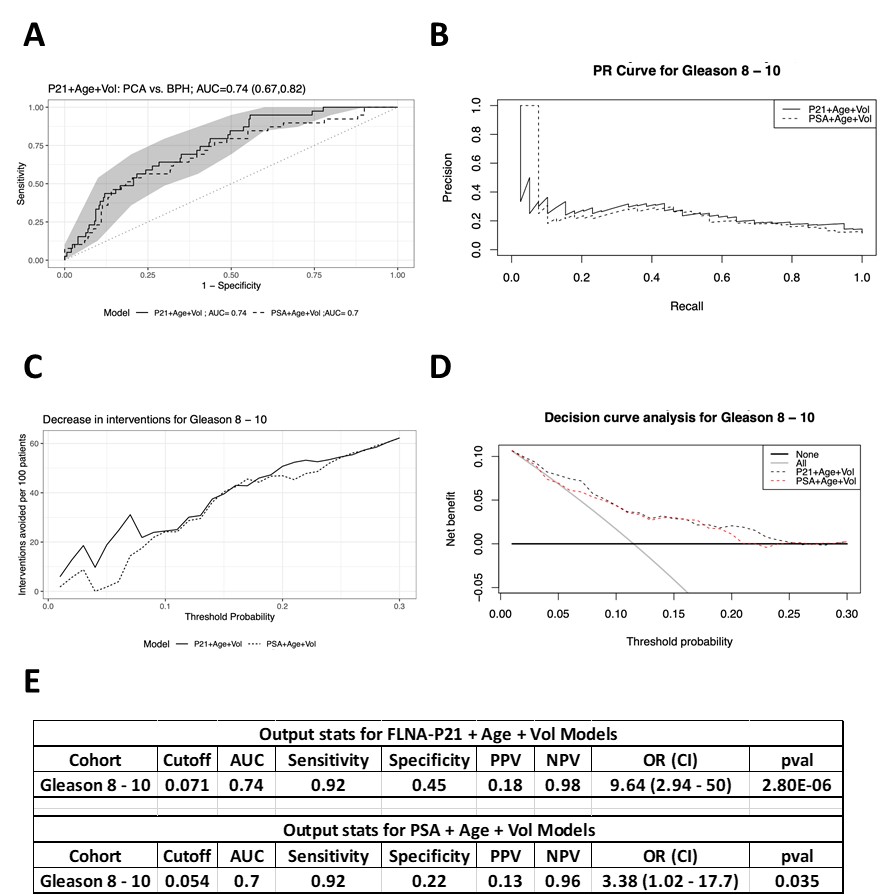


**Supplemental Figure 3:** FLNA Prostate Cancer Biomarker Panel Performs Better Than PSA combined with prostate volume and age.in detecting BPH/LUTS compared to Gleason 8-10 prostate cancers. **(A)** Combined ROC curve assessment. **(B)** Precision analysis, **(C)** Intervention analysis, and **(D)** decision curve analysis **(E)** statistical assessment comparing FLNA, prostate volume, and age with PSA, prostate volume, and age.
